# Adopting a blended learning approach to teaching evidence based medicine: a mixed methods study

**DOI:** 10.1186/1472-6920-13-169

**Published:** 2013-12-17

**Authors:** Dragan Ilic, William Hart, Patrick Fiddes, Marie Misso, Elmer Villanueva

**Affiliations:** 1 Department of Epidemiology & Preventive Medicine, School of Public Health and Preventive Medicine, Monash University, Level 6, The Alfred Centre, 99 Commercial Rd, Melbourne 3004, VIC, Australia; 2 Faculty of Health Sciences, Curtin University, Bentley, Australia; 3 Gippsland Medical School, Monash University, Churchill, Victoria, Australia; 4 Peninsula Health, Frankston, Victoria, Australia; 5 Monash Centre for Health Research and Implementation, School of Public Health & Preventive Medicine, Monash University, Melbourne, Victoria, Australia

**Keywords:** Evidence based medicine, Blended learning, Graduate medical education, Pedagogy

## Abstract

**Background:**

Evidence Based Medicine (EBM) is a core unit delivered across many medical schools. Few studies have investigated the most effective method of teaching a course in EBM to medical students. The objective of this study was to identify whether a blended-learning approach to teaching EBM is more effective a didactic-based approach at increasing medical student competency in EBM.

**Methods:**

A mixed-methods study was conducted consisting of a controlled trial and focus groups with second year graduate medical students. Students received the EBM course delivered using either a didactic approach *(DID)* to learning EBM or a blended-learning approach *(BL)*. Student competency in EBM was assessed using the Berlin tool and a criterion-based assessment task, with student perceptions on the interventions assessed qualitatively.

**Results:**

A total of 61 students (85.9%) participated in the study. Competency in EBM did not differ between the groups when assessed using the Berlin tool (p = 0.29). Students using the *BL* approach performed significantly better in one of the criterion-based assessment tasks (p = 0.01) and reported significantly higher self-perceived competence in critical appraisal skills. Qualitative analysis identified that students had a preference for the EBM course to be delivered using the *BL* approach.

**Conclusions:**

Implementing a blended-learning approach to EBM teaching promotes greater student appreciation of EBM principles within the clinical setting. Integrating a variety of teaching modalities and approaches can increase student self-confidence and assist in bridging the gap between the theory and practice of EBM.

## Background

Evidence Based Medicine (EBM) has been adopted as a core unit across many medical schools [1]. The principles of EBM inform medical decision making by integrating the best available evidence with the clinician’s clinical expertise and patient values [2]. Adopting an evidence based approach to medicine requires that users are competent in understanding and applying the following steps in clinical practice:

(i) Asking a clinical question that is constructed using the PICO (patient, intervention, comparison, outcome) framework;

(ii) Acquiring the evidence via a systematic and efficient search of the literature;

(iii) Appraising the evidence through the application of critical appraisal techniques;

(iv) Applying the evidence to the clinical scenario; and,

(v) Assessing the EBM process as it relates to the clinical context [2].

Each step within the EBM process requires a different level of knowledge and skill (i.e. competence) from the user [3]. Achieving a high level of competency in EBM can only be achieved when the user is able to effectively undertake all five steps, which incorporate adequate levels of knowledge, skills and behavioural elements [4]. Achieving competency in the principles of EBM can provide the user with the ability to achieve lifelong learning within the clinical setting.

Learning is influenced by a variety of factors including the student, teacher, course/curriculum and educational environment [5]. In creating a supportive educational environment, educators must consider the different learning styles preferred by students including; visual, auditory, kinaesthetic, procedural, or a combination of these [6]. Continuing medical education has traditionally been facilitated through the use of didactic lectures [7,8]. Recent educational research has shifted the focus on self-directed and adult educational pedagogies through a variety of delivery modalities (lectures, interactive workshops, practice-based interventions, problem-based learning and simulation through eLearning) for optimal educational outcomes [7-12].

Limited research has been conducted into evidence to inform the best method of teaching EBM. A 2004 systematic review identified two randomised controlled trials (RCTs) and seven non-RCTs that examined the impact of post-graduate teaching in EBM [13]. The authors concluded that that standalone teaching improved student knowledge, but not skills, attitudes or behaviour in EBM. Conversely, integrating teaching of EBM with clinical activities resulted in improvements across all four outcomes [13].

Few rigorous studies have explored methods of teaching EBM to medical students. A 2005 RCT evaluated computer-assisted self-directed learning with workshops in EBM with undergraduate medical students [14]. This study concluded no difference in student knowledge, skill or attitudes on EBM across the two interventions [14]. Conversely, a 2010 study with medical undergraduates assessed the integration of online learning of EBM skills with clerkships during the third year of study [15]. Using a before and after methodology it identified that student competency in EBM was significantly improved over the duration of the course [15].

A 2008 RCT explored the impact of teaching EBM using a computer-based approach compared to traditional didactic lectures to medical undergraduate students [16]. The study demonstrated equivalency in EBM knowledge and attitude scores between students who received the computer-based intervention compared to students receiving the course via didactic lectures [16]. These findings were also reflected in an early study with medical post-graduates exploring the same delivery modes [17]. More recently, a RCT demonstrated that teaching EBM via a case conference resulted in significantly higher knowledge and personal application of EBM related content in final year medical students, compared to those receiving the same information in a didactic format [18].

Over the last decade many medical schools have reduced the amount of didactic teaching and implemented a problem-based learning (PBL) approach to teaching clinical skills to medical students. Within this context, a PBL approach utilises authentic clinical queries from which students utilised their existing knowledge to explore and construct new knowledge, skills, attitudes and behaviours [19]. Implementing a PBL, or case-based approach, within a medical curriculum provides an opportunity for students to contextualise their learning within the clinical environment [19]. A 2009 RCT examined the effectiveness of delivering an EBM course to medical students using a PBL approach compared to usual teaching methods (lecture plus tutorial) [20]. This RCT identified that the PBL approach was less effective than usual teaching at improving student knowledge in EBM, but was more effective at increasing positive attitudes toward EBM [20].

An extension of utilising the PBL approach in teaching EBM is blended-learning. Utilising a PBL approach to teaching EBM attempts to add the element of ‘clinical realism’ to the case. Blended-learning, whereby the use of digital technology and other ‘non-traditional’ teaching methods are integrated to add greater flexibility to the teaching curriculum, but also account for differing learning styles exhibited by students [21,22]. Relatively few studies have empirically examined the effectiveness of blended-learning in medicine, with all studies focusing on the impact of blended-learning in a clinical discipline. Results of those published studies commonly report an increase in student satisfaction with the content, better use of time in class, increase in knowledge and promote self-directed learning [23-25].

Currently there is a lack of consensus within the medical literature as to the most effective method of teaching medical students the principles of EBM. The overall aim of this study was to identify whether a blended-learning approach to teaching EBM was more effective than a didactic-learning approach at increasing medical student competency in EBM. Student perceptions regarding the strengths and limitations of each mode of delivery were also sought.

## Methods

A mixed methods approach consisting of a controlled trial and focus group was adopted for this study [26].

### Study design and setting

A controlled trial with intention-to-treat analysis was performed with second year medical students undertaking the graduate MBBS degree at Monash University. The graduate MBBS degree is a four year graduate entry course delivered by the Gippsland Medical School (GMS). Students spend the first year of the course based at the Churchill campus of the GMS in which basic medical and behavioural sciences are taught, in addition to students participating in community partnership programs. In this year, students receive introductory lectures and tutorials on the principles of EBM and epidemiology. Students spend the second year of their degree in the clinical environment, spending their entire year based at only one clinical site (Traralgon, Warragul, Sale or Peninsula). It is during this second year in which a comprehensive EBM program is delivered to students. Students based at the first three clinical sites participate in ‘block’ days, in which educational content is delivered in a didactic format (i.e. discrete lectures and tutorials), problem-based learning and case-based learning sessions. Students based at the Peninsula site received the same educational content, delivered via a blended-learning approach. Students across all clinical sites were taught the same concepts of EBM, the only difference between the groups was the mode of delivery (Table 1). Both groups have 10 tow-hour sessions of EBM teaching.

**Table 1:** Overview of the EBM course content

**Session**	**Key EBM content covered**
1. Introduction to EBM	• Rationale for EBM in medicine
	• How to construct a clinical question
2. Searching the medical literature	• Overview of relevant medical and healthcare databases
	• How to construct a search strategy
3. Biostatistics	• Overview of biostatistical concepts including;
	▪ Categorical versus numerical data
	▪ Use of appropriate statistical analysis
4. Critical appraisal of studies of therapy (part 1)	• Introduction to RCTs
	• Measures of effect (relative risk, number needed to treat, absolute risk)
	• P-values and confidence intervals
	• Critical appraisal techniques for studies of therapy
5. Critical appraisal of studies of therapy (part 2)	• Continuation of session 4.
6. Critical appraisal of studies of harm (part 1)	• Introduction to cohort studies
	• Measures of effect (Odds ratios and number needed to harm)
	• Critical appraisal techniques for studies of harm (specific to cohort studies)
7. Critical appraisal of studies of harm (part 2)	• Introduction to case–control studies
	• Measures of effect (Odds ratios and number needed to harm)
	• Critical appraisal techniques for studies of harm (specific to case–control studies)
8. Critical appraisal of studies of diagnosis	• Overview of concepts specific to diagnosis including;
	▪ Sensitivity and specificity
	▪ Positive and negative predictive values
	▪ Positive and negative likelihood ratios
	• Critical appraisal techniques for studies of diagnosis
9. Critical appraisal of studies of prognosis	• Overview of concepts specific to prognosis including;
	▪ Longitudinal study designs (including time series)
	▪ Use of survival curves and hazard ratios
	• Critical appraisal techniques for studies of prognosis
10. Critical appraisal of systematic reviews	• Introduction to how systematic reviews are constructed
	• Overview of how to interpret meta-analysis including;
	▪ Forest plots
	▪ Sensitivity analysis
	▪ Significance of heterogeneity
	• Critical appraisal techniques for systematic reviews

### Recruitment

Second year graduate medical students were recruited from four teaching hospitals associated with the course (Traralgon, Warragul, Sale and Peninsula). In order to meet eligibility, participants were required to be a second year Monash graduate MBBS student at the time of the study. Students who were unwilling to participate in the study, or did not wish to provide consent, were excluded from the recruitment process.

### Allocation

**Figure 1:**
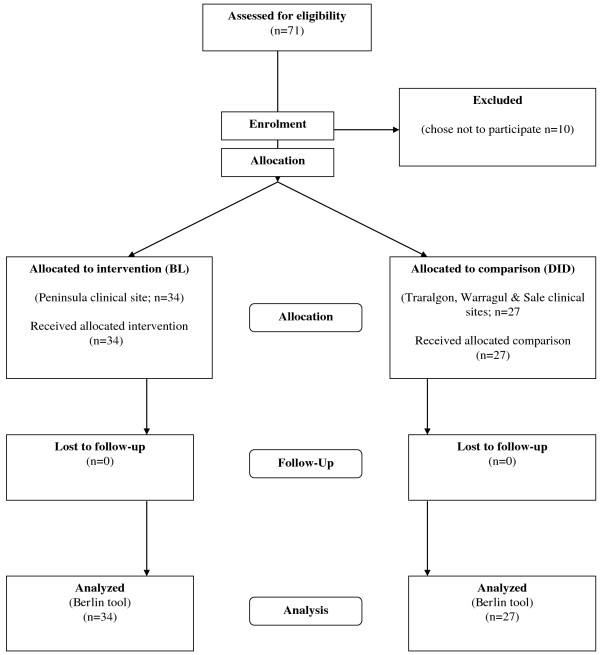
Flow diagram of trial.

In this study, students attending the Peninsula clinical site received the intervention, whilst students across the remaining sites (Traralgon, Warragul and Sale) received the same EBM content delivered via the existing method of delivery (Figure 1). The Peninsula site was chosen to receive the intervention due to convenience of delivering the content at one site, as opposed to the logistics of organising the intervention at three separate sites.

### Blended-learning *(BL)* EBM delivery

Students allocated to the blended-learning model received a one-day ‘block’ workshop, which covered all the EBM concepts that are delivered in the existing tutorial-based delivery of the EBM course. This ‘block’ workshop utilised two tutorial sessions worth of time. Students were directed to additional EBM content, accessible through the Monash University library website, to support self-directed learning. The remaining eight tutorials designated to EBM teaching were used in this group for students to present their patient-based EBM scenarios and generate discussion with the tutor, who in the *BL* approach acted as a facilitator rather than a tutor, in order to facilitate discussion within the group and promote peer to peer learning [27]. Peer to peer learning was facilitated through the use of a quasi-journal club delivery method [28]. At the beginning of each tutorial session, the facilitator would divide students into small groups, with each student given a specific health topic, or intervention/exposure, to investigate. Students were then required to identify a patient during their clinical rotation, for which the scenario would be applicable. Students were required to take a detailed medical history from the patient, adopt the principles of EBM and identify and critically appraise an article on the topic that could be applied to the patient. At the following tutorial session students were required to present their patient and related EBM content as part of a patient-based presentation.

### Didactic *(DID)* EBM delivery

Students allocated to this group received the EBM course delivered via a didactic-learning approach, which is the existing mode of delivery for the course. In this version of the EBM course, students attend 10 two-hour tutorial sessions. An outline of the EBM course is presented in Table 1. All of the sessions begin with the tutor providing a short presentation on the relevant EBM concept for the session. This is followed by students completing small group tasks and participating in large group discussions, based on the teaching materials, with the tutors leading the discussion. Tutors in the *DID* group led the tutorial with structured activities and were therefore classified as ‘tutors’, rather than promoting peer learning and facilitating discussion within the group as per the ‘facilitators’ in the *BL* group.

### Outcome measures

The primary outcome measured in this study was competency in EBM. Competency in EBM was measured using the previously validated Berlin tool [29]. The Berlin tool consists of 15 multiple choice questions, which assesses knowledge and skills in EBM. The maximum score on the Berlin tool is 15. During the EBM course, students complete two criterion-based course assessment tasks, which assesses student competency across the first four steps of the EBM continuum. Both tasks require students to (i) identify an appropriate clinical scenario, (ii) based on the scenario, construct a clinical question, (iii) identify an appropriate study from the literature to answer the question, (iv) critically appraise the article, (v) implement the findings to their clinical scenario). The first assignment is based on a ‘therapy’ scenario, whilst the second assessment task is based on a ‘harm’ scenario. Both assessment tasks are criterion-based, with a final score calculated out of 100%. Both assignments were graded by EBM tutors participating in this study based on a previously developed rubric. No psychometric testing of the marking rubric was performed. All outcome measures were assessed at the conclusion of the respective EBM courses. Students also completed a questionnaire that assessed their self-perceived competence across the various EBM skills and attitudes toward the course. All questions were measured on a five-point Likert scale (1 = strongly disagree, 5 = strongly agree). The questionnaire was specifically developed for this study, but did not undergo any psychometric testing. All outcomes were assessed at the conclusion of the EBM program during the second year of the graduate program.

### Qualitative data collection

A phenomenological approach to collecting qualitative data, through the use of focus groups, was adopted to identify student perceptions on the delivery of the EBM course using the existing *DID* versus the *BL* learning approach [30]. Collection of qualitative data through focus groups provides a collective perspective on the topic of interest, and facilitated quick access to a larger sample compared to in-depth interviews [31]. Focus groups were conducted with students across all four clinical sites at the conclusion of their respective EBM courses. All students were recruited via convenience sampling through a bulk email sent to each clinical site at the conclusion of the EBM teaching program. Students interested in participating in a focus group were required to contact the clinical site administrator, who then organised a suitable time and date. Each focus group consisted of between six to eight students per clinical site. All students volunteered to participate in the focus groups and were not paid for their contribution. The two focus groups at the Peninsula clinical site were moderated by the same facilitator (DI), an experienced facilitator who is also the coordinator of the EBM course at Monash University. A semi-structured interview guide was developed from a review of the literature before the commencement of focus groups. The use this guide ensured that all discussion points were consistent across the focus groups. The remaining three focus groups across the Traralgon, Warragul and Sale were facilitated by an independent researcher, using the same discussion points as used in the Peninsula focus groups.

### Data analysis

Quantitative data were assessed for Normality before analysis. Difference in EBM competency based on the Berlin tool and the assessment tasks was assessed using the two-tailed, non-parametric Mann–Whitney U test. Differences in student self-perceived competency in EBM, and attitudes toward EBM, was also assessed using the two-tailed, non-parametric Mann–Whitney U test. All focus groups were audio-taped with a digital recorder, downloaded onto computer and transcribed verbatim by an administrator within the Department of Epidemiology & Preventive Medicine. All transcripts were de-identified to preserve the anonymity of participants. All transcripts were analysed independently by two investigators (DI and MM) using the principles of thematic analysis, with the assistance of the NVivo program [30]. Themes were identified by coding features of the data, then collating into relevant themes, before finalising the specifics of each theme [30]. Both investigators independently coded and categorised emerging themes from the data, before a consensus on the overall themes was reached.

### Ethics

Ethics approval for this study was received by the Monash University Standing Committee on Ethics in Research Involving Humans.

## Results

From a total of 71 eligible students, 61 (85.9%) participated in this study. All 61 students completed the quantitative assessment of the study, with 37 students participating in the focus group discussions (15 from Peninsula, seven from Sale, seven from Traralgon and eight from Warragul). No statistical difference in EBM competency was identified between students allocated to the *DID* group versus the *BL* group when using the Berlin tool (p = 0.29) (Table 2). When using the course assessment tasks to evaluate student competency in EBM, it was identified that that no significant difference existing between the groups when undertaking the first assessment task (p = 0.14). However, students allocated to the *BL* group scored a significantly higher grade on the second course assessment task when compared to the *DID* (p = 0.01).

**Table 2:** Assessment of student competency in EBM using the Berlin tool and a criterion-based course assessment task

**Assessment tool**	***BL *** **(n = 34)**	***DID *** **(n = 27)**	**P-value**
Berlin tool (mean score (95% CI))	6.08 (5.18-6.99)	6.77 (5.51-8.04)	0.29
**Assessment tool**	***BL *** **(n = 36)**	***DID *** **(n = 35)**	**P-value**
Assessment Task 1 (mean percentage (95% CI))	93.65 (90.57-96.72)	95.14 (93.42-96.86)	0.19
Assessment Task 2 (mean percentage (95% CI))	97.43 (95.43-99.43)	96.00 (94.24-97.76)	0.01

Students in the *DID* group had a higher self-perceived competence in completing step 1 (constructing a clinical question) and step 2 (searching the literature) of the EBM process in comparison to the *BL* group (Table 3). Conversely the *BL* group had a significantly higher self-perceived level of competence when completing step 3 of the EBM process (critical appraisal of studies). Students allocated to the *BL* group were significantly more satisfied with the delivery of the EBM course, finding it to be more stimulating and applicable to their clinical studies (Table 3). These students also had a greater belief that they would use their EBM skills during their clinical career.

**Table 3:** Student self-perceptions about EBM competency and attitudes about EBM

**Question**	***BL *** **(n = 27) (mean score (95% CI))**	***DID *** **(n = 34) (mean score (95% CI))**	**P-value**
1. I can confidently construct an answerable question using the PICO framework	4.0 (3.82-4.17)	4.34 (4.04-4.64)	0.01
2. I can conduct an effective literature search using MEDLINE	4.06 (3.82-4.17)	4.18 (3.90-4.46)	0.39
3. I understand how biases (selection, performance, attrition, detection) may affect the validity of a study	3.44 (3.20-3.68)	3.62 (3.40-3.85)	0.31
4. I can confidently calculate and interpret different measures of effect (i.e. RR, RRR, ARR, NNT)	3.31 (3.02-3.60)	3.43 (3.13-3.74)	0.58
5. I can confidently critically appraise studies of ‘therapy’ and apply the findings to a clinical context	3.89 (3.65-4.10)	3.75 (3.50-3.99)	0.39
6. I can confidently critically appraise studies of ‘harm’ and apply the findings to a clinical context	3.86 (3.64-4.08)	3.50 (3.25-3.74)	0.04
7. I can confidently critically appraise studies of ‘diagnosis’ and apply the findings to a clinical context	3.82 (3.62-4.03)	3.34 (3.12-3.56)	0.01
8. I can confidently critically appraise studies of ‘prognosis’ and apply the findings to a clinical context	3.72 (3.49-3.94)	3.34 (3.12-3.56)	0.01
9. I can interpret a systematic review and apply the findings to a clinical context	3.79 (3.55-4.02)	3.46 (3.17-3.75)	0.05
10. This unit enabled me to achieve its learning objectives	3.72 (3.45-3.99)	3.43 (3.13-3.74)	0.12
11. I found the unit to be intellectually stimulating	3.72 (3.43-4.01)	3.03 (2.69-3.36)	0.01
12. Overall I was satisfied with the quality of this unit	3.69 (3.36-4.01)	3.18 (2.84-3.53)	0.03
13. I have used my EBM skills when studying this year	3.31 (2.97-3.64)	2.78 (2.44-3.12)	0.02
14. The workload for each EBM session was reasonable	3.93 (3.64-4.21)	3.62 (3.28-3.96)	0.22
15. I believe that I will use my EBM skills during my clinical career	4.20 (3.97-4.44)	3.81 (3.54-4.07)	0.03
16. I believe that practicing evidence based medicine is critical in being a good clinician	4.44 (4.20-4.68)	4.12 (3.85-4.39)	0.08

**Figure 2:**
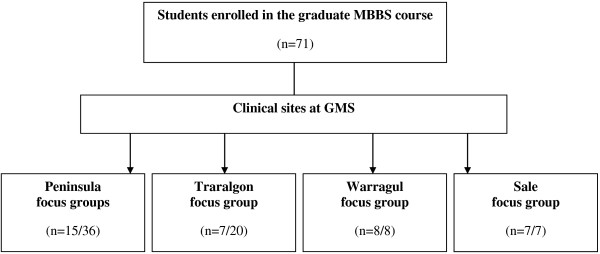
Structure of focus groups across clinical sites, with number of students participating in focus groups.

A total of 37 (52.1%) students participated in focus group discussions (Figure 2). Two focus groups were conducted with students based at the Peninsula clinical site (which ran the *BL* program) and three focus groups at the Traralgon, Warragul and Sale clinical sites (which ran the *DID* program). The following themes emerged from the focus groups. Themes emerging from the focus group discussions centred on the use of blending learning in EBM, role of tutors and librarians, assessment and use of EBM as future clinicians.

### BL students

#### Using a blended-learning approach

Students preferred using a blended-learning approach as it primarily allowed them to make the link between the theoretical aspects of EBM and the practical aspect of application at the bedside with patients. The blended-learning approach was also perceived by students to place greater emphasis on student self-directed learning, whilst drawing on knowledge that was gained during the initial ‘block’ learning of EBM concepts. Students also perceived that tutorials were more interactive than those previously experienced in the EBM course due to students taking a greater ownership of presenting materials and research.

“I like that we got to base the research on a real patient. We got to dissect the different aspects as they relate to EBM and come up with a relevant topic that we could discuss.”

“The presentations each week that the (facilitator) provided were pretty good as well. It means that you’re doing your own research, and when you do that you kind of consolidate what you’re learning.”

“The (patient) case-based learning presentations are very interactive - you’re applying it as you learn it (on the ward).”

Students also identified limitations to using the blended-learning approach. Whilst students enjoyed the freedom of self-directed learning and researching new topics, a perceived lack of previous teaching on certain topic areas was perceived as a barrier preventing students from understanding the topic in depth. Students saw the tutorial time as a lost opportunity for the facilitators to ‘teach on the run’ and fill in the knowledge gaps when they arose on such topics.

“Something I found really frustrating was that you’d get this topic and you’d go home and look it up, and you’d start looking into it and you’d realise that it didn’t make sense, because we didn’t know anything about it.”

“I think the sessions are good, but it would also be good to use the time to go through certain concepts.”

#### Block learning

Students demonstrated a preference of ‘block’ learning critical information at the beginning of the EBM course. The ‘pre-loading’ of theoretical EBM concepts, followed by the blended-learning tutorials, was viewed by students as the preferred mode of delivering an EBM course.

“I didn’t mind having it all on the one go at the start: It was good, it allows us to consolidate all the content in a day, and then hopefully apply it throughout the year.”

Whilst students demonstrated a preference for the block learning approach, students also identified that there was a need to better utilise tutorial time to revisit and further discuss certain EBM concepts in order to consolidate student knowledge.

“I think we were doing refresher tutes throughout the year as well. Because sometimes I feel I’m out of touch with certain concepts and equations.”

#### Role of the tutor/facilitator

Students were pleased with tutors taking a role in which they acted as facilitators for the session, rather than traditional tutors who might present the teaching materials in a didactic format. Students also demonstrated a preference for the facilitators to have some form of a clinical background, regardless of whether they were still practicing clinicians or had clinical experience. Students also demonstrated a preference for facilitators to be ‘experts’ in the field of EBM.

“I think it would be good to have clinicians because they have been through the process of searching journal articles to look up for the latest treatments and all that. So they make the teaching more relevant to us, in a sense.”

“When you’re presenting the case and you’re trying to form a question, you really want someone that knows exactly what goes on.”

Whilst the students preferred the use of facilitators, there was a perceived need for greater direction to be provided by facilitators at the beginning of the session. Such instruction was important to students given that the sessions were predominantly led by the student-directed research on their respective case.

“It would be useful for them (the facilitators) to help us read up on what might be appropriate on the topic, then whoever is presenting for the week would know that they have to go and find a patient who has a thyroid issue or whatever it may be…”

### DID students

#### Using the didactic approach

Students presented conflicting viewpoints when asked whether they preferred the EBM content to be delivered in the existing tutorial format, or using the proposed blended-learning approach. Much of this difference was dependent on the preferred learning style of students. Whilst the tutorials are intended to be interactive in their design, the majority of tutorials seem to incorporate a large amount of didactic teaching of EBM concepts. Students that preferred a didactic approach to learning demonstrated a preference for EBM concepts to be delivered in the existing tutorial format. Conversely, other students perceived that the tutorial style of teaching did not consolidate, or build upon their previous knowledge and skills in EBM. These students voiced that the course was subsequently not stimulating from a clinical viewpoint.

“The presentations, I thought, were taught better than they were last year. They actually made sense. Last year was all jumbled up, but I thought this year was a bit more structured, with what we were supposed to get out of it.”

“The way in which they (the tutors) were delivering the material was boring. It didn’t seem to me like here were two people who had sat down and thought ‘how can we best deliver this material?’ It was like, ‘well, here are the slides, we’ll read through them and deliver the material.’ I think if they’d used the two hours – like, if every week we’d had that two hours used more effectively, we would have been really, really strong in this subject, and I don’t think we are.”

#### Block learning

Students demonstrated a desire for consolidating their learning using a ‘block style’ approach. Students discussed that the EBM course could be effectively taught in a large group, in which critical concepts in EBM were introduced and then further discussed when students broke back into the original tutorial groups. This description essentially mirrored the ‘block’ learning approach delivered to the *BL* group.

“Look, we go back to ‘back to base’, where we get clumped up into a bigger group. I would be more than happy to study this subject in a bigger group, with one expert in EBM teaching. I’d love it. It would be better over this eight people in a broken up group with somebody who can’t teach it.”

#### Role of the tutor

Students in the *DID* group did not demonstrate a preference for tutors to be solely clinical experts, over content experts in EBM. Rather, students placed greater emphasis on the tutor being able to demonstrate the integration of EBM with clinical practice.

“You have lawyers come and try and teach us law, that’s not appropriate. But if you have lawyers who know their topic very well and understand that they’re giving it to medical students, it’s still really useful. The same with this (EBM).”

### Common themes across both groups

#### Use of a dedicated library session

Both cohorts participated in a two-hour library session in which the clinical site’s subject librarian presented an overview of relevant EBM databases and methods of constructing an effective search strategy. Both student cohorts found that the library session was practical and useful – particularly for techniques for accessing quick evidence-based information in the clinical setting.

“The librarian actually taught us how to use stuff we needed to know… that, sort of practical ‘how do you go about doing it? type of stuff.” (DID group)

#### Assessment tasks

Both cohorts demonstrated positive opinions about the use and value of the assessment tasks. Both groups believed that the assessment tasks were a valid tool in assessing student competency in EBM.

“They’re (the assessment tasks) pretty comprehensive; you’ve got to cover a lot. It’s good to know the ins and outs of assessing articles and knowing whether they’re good or not.” (BL group)

“The assignments tested what we were supposed to be taught very well.” (DID group)

#### The use of EBM as clinicians

Both groups were asked whether they would use the EBM skills taught in the future as clinicians. Whilst students did not explicitly use the skills currently as students, for example during the study, both cohorts believed that they would use EBM skills in the future as clinicians.

“I think it’s an essential part of being a clinician. It’s kind of what separates us from quacks – to be able to critically appraise evidence, and also to use those tools to further medicine as well.” (DID group)

“As we’re specialising, and trying to keep up to date with all the different things, that’s when we’ll use it the most – to see if this new information is valid or not.” (BL group)

## Discussion

This study generates novel findings on the impact of adopting a blended-learning approach to EBM in graduate-entry medical students. Our findings also demonstrated no difference in EBM competency between students who received a traditional didactic, tutorial-based implementation of an EBM course compared to a blended-learning approach. Conversely, it identified that students prefer utilising a blended-learning approach to learning EBM as it is perceived to offer a greater opportunity to integrate the theoretical concepts of EBM with the practical situations of clinical practice.

Findings from this study concur with those of a systematic review that concluded that standalone teaching may only improve knowledge, but not attitudes, skills and behaviour in EBM in postgraduate students [13]. Similarly, it provides further evidence that utilising a PBL approach to EBM may increase student attitudes and behaviour towards adopting the principles of EBM in clinical practice [20]. Students exposed to the *BL* approach found the EBM unit more intellectually stimulating, were able to translate their EBM skills to other components of their study and appreciated the link between theory and practice.

If an evidence-based approach to medicine is to be practiced by clinicians, then these future clinicians need to be taught how to use EBM as students during their clinical years. Providing evidence, be it physically or the tools to effectively search, identify, evaluate and implement, to busy clinicians increases the extent to which evidence is sought and incorporated into medical decision making [32]. Integrating EBM teaching alongside bedside and other PBL and blended-learning approaches provides students with an opportunity to improve competence in both their EBM and clinical skills – a nexus that it essential if EBM is to be applied in the clinical setting.

EBM has been criticised as ‘cookbook’ medicine and something that can only be practiced by those in ivory towers [33]. The principles of EBM rely on the integration of evidence, clinical expertise and patient values – all of which will differ across clinical scenarios. Studies have also demonstrated that clinicians, who practice their EBM skills in their limited downtime, can incorporate evidence and practice EBM in ‘real-time’ [33]. The proportion of clinicians incorporating and practicing EBM in their daily clinical workload varies considerably [34,35]. Barriers to successful implementation as practicing clinicians may include a lack of time, resources, patient-related factors or influence of peers [36]. Providing medical students with the knowledge and skills in EBM increases their ability to implement such skills in the clinical setting [37]. It remains uncertain whether the influence of the above mentioned barriers negates the transfer of their EBM skills in clinical practice.

This study demonstrates that adopting blended-learning approach to teaching and learning EBM provides a framework that integrates with the existing steps of the EBM process. The blended-learning approach is clinically focused, with the problem-based aspect encouraging learners to rely on their existing EBM knowledge whilst implementing their EBM skills to identify, evaluate and implement evidence relevant to the clinical scenario. This approach demonstrates to medical students at an early clinical phase of their education that EBM is not ‘cookbook’ medicine, but a lifelong tool that can be applied in the clinical environment [38,39].

### Study limitations

The principles of EBM place the RCT as the ‘gold’ standard since in study design since many methodological issues including selection, performance, attrition and detection biases may be controlled. This study was not a RCT, but a pragmatic trial, since it was not possible to randomise and blind individual students to the intervention. The use of a mixed methods approach, integrating quantitative and qualitative data further contextualised and triangulates the results of this study. This study has demonstrated the effectiveness of adopting a blended-learning approach to teaching EBM. This blended-learning approach was successfully implemented in a small teaching hospital. The feasibility of implementing this approach in a large teaching hospital remains uncertain. Student numbers will dictate how many facilitators are required, of which few seem to have both the clinical and EBM expertise so often desired by students.

DI is the coordinator of the EBM program, but also facilitated the focus group discussions. This raises the possibility that this dual role may influence the manner in which students express their perceptions about the *BL* and *DID* learning styles. During the recruitment and conduct of the focus groups, it was strongly reiterated that participants may openly express any views on the EBM course; which would seem to be reflected in the responses provided. Assess of EBM competency was assessed by the Berlin tool, which has been previously validated and psychometrically tested for this purpose. Both the assessment tasks and self-reported perception questionnaire have not been psychometrically validated.

## Conclusions

The findings from this study suggest that a blended-learning approach to teaching EBM promotes greater student appreciation and increase in self-confidence in using the EBM principles within the clinical setting. This direct application to the clinical environment provides an opportunity to bridge the gap between theory and practice. Future research is required to investigate whether similar findings are apparent in undergraduate-based medical students and the feasibility of implementing such a program among a large student cohort.

## Competing interests

DI is the coordinator of the EBM program for the MBBS degree at Monash University. PF and EV coordinate the EBM teaching program delivered through the Gippsland Medical School.

## Authors’ contributions

DI designed the study, collected the data, performed quantitative and qualitative data analysis and drafted the manuscript. WH contributed to the design of the study, and drafted the manuscript. PF contributed to the design of the study, and drafted the manuscript. MM performed the qualitative data analysis and drafted the manuscript. EV designed the study, collected the data, performed quantitative data analysis and drafted the manuscript. All authors read and approved the final manuscript.

## Authors’ information

DI is an Associate Professor in Evidence Based Clinical Practice at the School of Public Health & Preventive Medicine, Monash University.

WH is Professor Hart is the Foundation Head of Medicine at Curtin University.

PF is the Director of Undergraduate Clinical Education and Clinical Training at Peninsula Health and Adjunct Associate Clinical Professor Monash University.

MM is the Head of the Evidence Synthesis Program at the School of Public Health & Preventive Medicine, Monash University.

EV is an Associate Professor in Public Health and the Director of Research at the Gippsland Medical School, Monash University.

## Pre-publication history

The pre-publication history for this paper can be accessed here:

http://www.biomedcentral.com/1472-6920/13/169/prepub
